# Development of split-root assays for loblolly pine (*Pinus taeda* L.) seedlings to study ectomycorrhizal symbioses

**DOI:** 10.1016/j.mex.2023.102046

**Published:** 2023-02-05

**Authors:** Benjamin D. Rose, Hannah E.R. Frank, Kevin Garcia

**Affiliations:** Department of Crop and Soil Sciences, North Carolina State University, Raleigh, NC 27695, United States

**Keywords:** Ectomycorrhizal symbiosis, Hebeloma cylindrosporum, Hydroponics, Loblolly pine, Paxillus ammoniavirescens, Split-root, Split-root assays for loblolly pine

## Abstract

Split-root techniques are valuable to investigate systemic vs*.* local plant responses to biotic and abiotic environmental factors, including interactions with soil microbes. Loblolly pine (*Pinus taeda* L.) is an economically important tree species that associates with many ectomycorrhizal fungi. However, a protocol for the establishment of split-roots experiments with loblolly pine has not been described so far. This method successfully establishes a split-root system in eight weeks following germination of loblolly pine seedlings. Rapid lateral root elongation is promoted by cutting the primary root tip and growing the seedlings in a hydroponic medium. Lateral roots can then be divided into two separated compartments and inoculated with ectomycorrhizal fungi. The method was validated by growth of split roots with or without inoculation. Root dry biomass was not significantly different between separated non-inoculated roots. Ectomycorrhizal colonization was not detected on the non-inoculated side of roots that were inoculated only on one side, demonstrating the success of the technique as a valuable method for split-root experiments in *P. taeda*. In addition to ectomycorrhizal fungi, researchers can use this method with loblolly pine to study systemic and local responses to a variety of other biotic or abiotic factors in the root environment.

• We describe a protocol to produce split-roots in loblolly pine (*Pinus taeda* L.) in eight weeks.
• This protocol uses hydroponics to promote the elongation of loblolly pine roots.
• We validated this protocol by determining split-root biomass and inoculating the seedlings with the ectomycorrhizal fungi *Paxillus ammoniavirescens* or *Hebeloma cylindrosporum*.

## Graphical abstract

A graphical abstract has been submitted along with this document.

**Figure:**
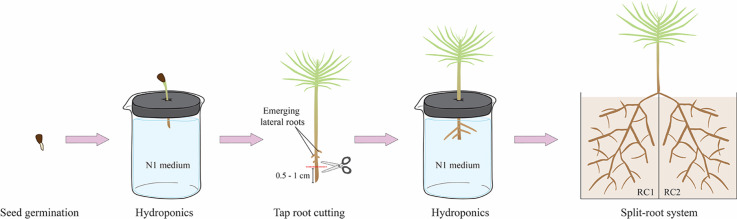

Specifications table

**Table:** 

Subject area:	Agricultural and Biological Sciences
More specific subject area:	*Root physiology and plant-microbe interactions*
Name of your method:	*Split-root assays for loblolly pine*
Name and reference of original method:	*N/A*
Resource availability:	*Reagents and Equipment are listed in the Materials section*

## Background

Plant roots interact with the soil and its biotic and abiotic components, making crucial the development of techniques to study these interactions. Split-root systems have been developed in multiple plant species and used to distinguish systemic versus local responses of roots to various environmental conditions, including resource availability, resistance to pathogens, or interactions with symbiotic microbes [1,2]. In split-root experiments, the roots of a single plant are divided into two relatively equal parts and grown in separate and distinct environments. Each root half can then be exposed to unique experimental treatment conditions. By measuring, in one root part, the effects of a treatment applied to distant roots of the same plant, researchers can discern between systemic and local signalling triggered by external soil factors.

Loblolly pine (*Pinus taeda* L.) is an important tree crop species originating in the Southeastern United States. Loblolly pine occupies approximately 11.7 million hectares of intensively managed forest land within its native range, in addition to an expanding worldwide distribution of exotic commercial plantations [3]. Loblolly pine forms beneficial symbiotic associations with a variety of ectomycorrhizal (ECM) fungal species that play a major role in the tree hydromineral nutrition [4], [5], [6]. Split-root experiments have been used in other pine species to study interactions amongst ECM fungi, host trees, and soil conditions [7], [8], [9]. However, to this date, no method for the rapid establishment of a split-root system for studying ECM symbioses in loblolly pine has been described. In fact, an extensive search of the literature found only one report describing loblolly pine in a split-root experiment studying plant physiological responses to soil temperature [10], but the seedlings were not grown in symbiosis with ECM fungi.

Here, we describe a method for establishment of split-roots with *P. taeda* in ECM symbiosis. The primary root tip was severed one month after seed germination, then seedlings were grown in hydroponic conditions for two months to encourage sufficient elongation of lateral roots for easy division into equal parts and inoculation in separate chambers. Allowing one month for ECM colonization and an additional month for response to treatment conditions, a complete split-root experiment to study ECM symbiosis can be carried out in approximately 5 months using this method. Although ECM symbiosis is the focus of the method as described here, this split-root technique can also be used to investigate the systemic or local responses of *P. taeda* roots to a variety of other treatments, including nutrient availability, abiotic stresses, pathogen tolerance, or interaction with other root-associated beneficial microbes.

## Materials

(1) 60 mm x 15 mm sterile plastic Petri dishes (Corning™ Falcon™ #351,007)
  Forceps, Fisherbrand™ filter/membrane forceps or similar (Fisher Scientific #09–73–50)
(2) Scissors, Fisherbrand™ utility scissors or similar (Fisher Scientific #08–945)
(3) 250 ml glass beakers (Kimax, USA, No. 14,000)
(4) Clone collars (Growneer®, diameter 2.75 in [6.985 cm], thickness 0.75 in [1.905 cm], 8 spokes). Make sure that clone collars will fit tightly into top of 250 ml beakers
(5) 1000 µl pipette tips with filters (USA Scientific, 1122–1830), sterile
(6) Aluminium foil
(7) Paper tape
(8) Plastic planting trays (Yield Lab,black plastic propagation tray, 25.4 cm x 50.8 cm x 5.5 cm; L x W x H)
(9) Plastic domes (Yield Lab propagation and humidity vented domes, 17.8 cm height, to fit planting trays)
(10) Plastic planting boxes (12 cm × 8 cm x 8 cm; L x W x H; from Carno A/S). [Six 5 mm holes drilled in the bottom of each pot to allow water uptake and drainage.]
(11) Stainless steel micro-spatula with rounded ends (Supertek Scientific #CH12218A), sterile
(12) Stainless steel cork borer (Cole-Palmer® #0,629,890, 1 cm internal diameter), sterile
(13) Culture jars for propagating fungal inoculants (DWK Life Sciences DURAN™ glass bottles with PP screw cap, Fisher Scientific #09–841–174 or similar)
(14) 100 ml glass beakers (Fisherbrand™, Fisher Scientific #FB100100), autoclaved
(15) 50 ml glass beakers (Fisherbrand™, Fisher Scientific #FB10050), autoclaved
(16) Immersion blender: Fisherbrand™ 150 Homogenizer with autoclaved stainless steel grinding tip probe (Fisher Scientific #15–340–167 & #15–340–178)
(17) SafeT-Sorb™ substrate (Ep Minerals® #7941) for potting medium
(18) Plastic coffee stir rods (KBG, 3-hole disposable coffee straw, 17 cm), sterilized under UV for 30 min each side, and cut to 12.5 cm (for supporting seedlings after transplant)
  Cable ties (TANTII® Industrial zip ties, 20.32 cm) sterilized with 90% ethanol (to tie seedling shoots to upright supports)
(19) 10 ml pipette tips (USA Scientific, 1051–0360), sterile

## Method details

### Plants and ECM fungi

*P. taeda* L. seeds were purchased in bulk from Sheffield's Seed Co. Inc., Arkansas, USA (Lot # 1,830,528). Seeds were kept at 4 °C prior to germination. For each experiment described below, 140 seeds were randomly selected.

Cultures of the ECM fungal species *Paxillus ammoniavirescens* Pou09.2 [11] and *Hebeloma cylindrosporum* Romagnesi h7 [12] were maintained on modified Melin-Norkans (MMN; [13]), and yeast-malt-glucose (YMG; [14]) agar media, respectively.

### *Pinus taeda* seed sterilization and germination

1. In a biosafety cabinet, place *P. taeda* seeds in an autoclaved 100 ml glass beaker with 50 ml 35% hydrogen peroxide (H_2_O_2_).
  Soak seeds in H_2_O_2_ for 15 min for surface sterilization.
  Remove H_2_O_2_ from the beaker using a sterile pipette.
  To rinse any remaining H_2_O_2_ from the seed surface and beaker, cover seeds with autoclaved milli-Q water and allow to soak for 10 min.
2. Remove the rinse water from the beaker using a sterile pipette.
3. Repeat this rinsing process (steps 4–5) for a total of 5 times.
4. After removal of the 5th rinse, cover seeds with autoclaved milli-Q water and cover the beaker with autoclaved aluminium foil.
5. Keep seeds at 4 °C for 72 h.
6. In a sterile biosafety cabinet, place sterilized seeds in Petri dishes containing agar (15 g·l^−1^) and glucose (2 g·l^−1^). Place 9 seeds equidistant in a line along the centre of each Petri dish. Take care to orientate the seeds such that roots will emerge in the same direction (Fig. 1A).

**Fig. 1:**
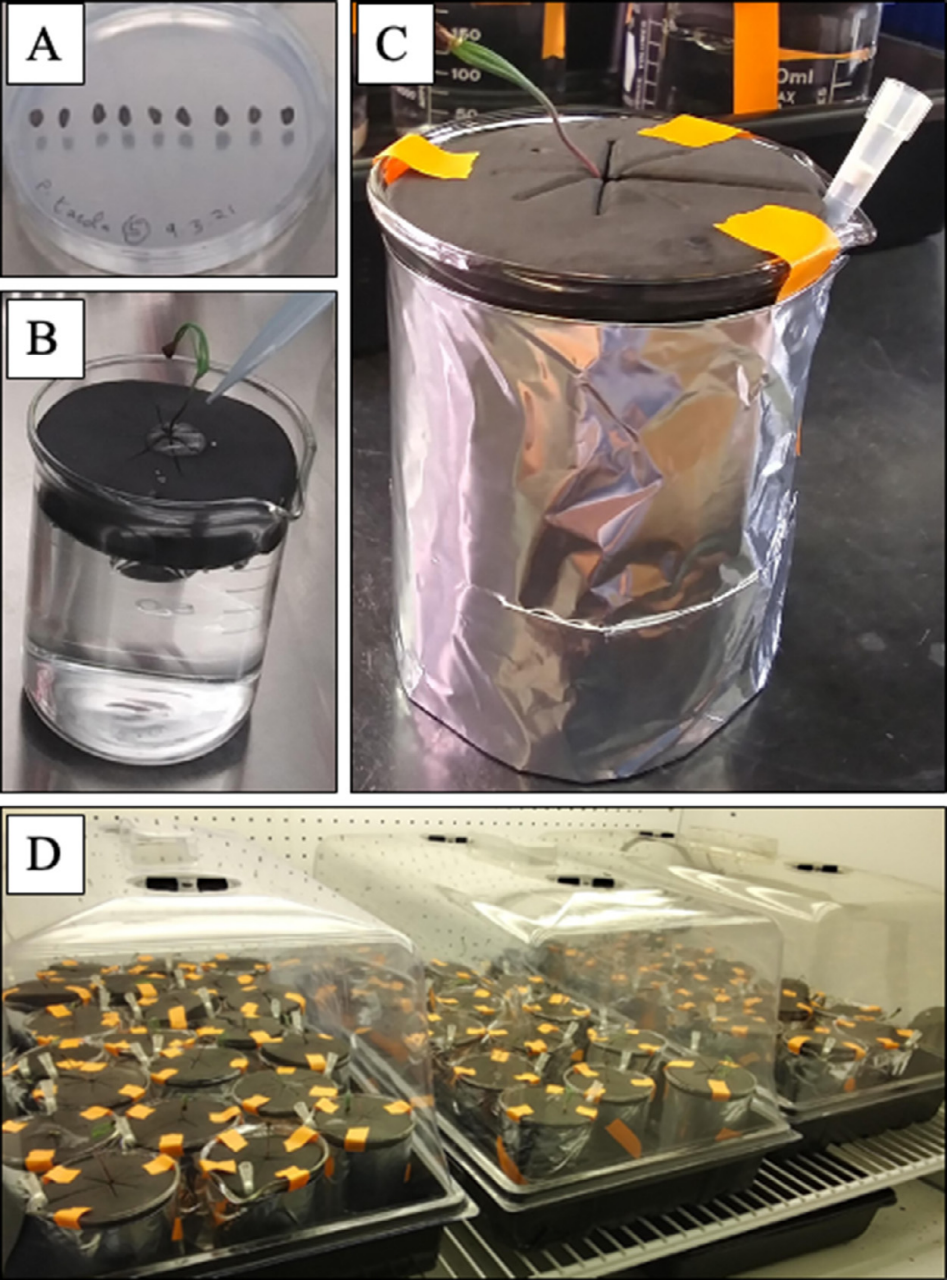
Preparation of individualized hydroponic systems for *Pinus taeda* seedlings. A. Sterilized seeds of *P. taeda* were placed in Petri dishes containing agar (15 g·l^−1^) and glucose (2 g·l^−1^). Dishes were then placed in a growth chamber (16 h day, 8 h night, 23°C, luminosity 210 µmol·m^−2^·s^−1^) for three weeks until germination. B. Germinated seedlings were transferred in sterile clone collars placed in 250 ml beakers filled with N1 nutrient solution. Then, 1 ml of N1 nutrient solution was applied to the base of the seedling shoot at the top of the clone collar. C. Clone collars were secured with paper tape, and a sterile 1000 µl filtered pipette tip was placed between the clone collar and inner surface of the beaker's spout for gas exchange.  D. Beakers were placed in a growth chamber (16 h day, 8 h night, 23°C, luminosity 210 µmol·m^−2^·s^−1^) for one to two weeks until the emergence of lateral roots.
7. Seal Petri dishes containing seeds with Parafilm and place in a tray upright at an approximate 45° angle.
8. Place trays in a controlled growth chamber (16 h day, 8 h night, 23 °C, luminosity 210 µmol·m^−2^·s^−1^). Maintain relative humidity at approximately 60%.
  Allow seeds to germinate in the growth chamber for 3 weeks.

### Preparation and sterilization of hydroponic materials and nutrient solution

1. Prior to seedling transplant, sterilize one clone collar and one 250 ml beaker per seedling:
  a. Open rubber foam clone collars slightly at perforations and submerge in 90% ethanol for 10 min to sterilize.
  b. Remove collars from ethanol and place upright in sterile biosafety cabinet, with open perforations facing upward, under UV light for 1 h.  After 1 h, lay collars flat to allow UV exposure on one side for 1 h, then turn over to expose the other side under UV for an additional 1 h.
  c. Gently squeeze each collar to check that ethanol has completely evaporated from pores in the foam rubber. Sterilized collars can be kept in a covered autoclaved container until transplant if necessary.
2. In the same biosafety cabinet, add 200 ml of autoclaved N1 nutrient solution (Table S1, [15]) to each autoclaved 250 ml glass beaker.

### Seedling transplant into hydroponic systems

1. Working in a biosafety cabinet, hold one clone collar open slightly and carefully remove a germinated seedling from the Petri dish using sterile forceps.
2. Place the seedling in the clone collar such that the transition zone between root and shoot is held in the centre of the clone collar opening, and root and shoot tissues extend beyond the upper and lower surface of the clone collar, respectively (Fig. 1B).
3. Close the clone collar around the seedling and insert the collar into the top of a 250 ml beaker filled with N1 nutrient solution.
  a. Evenly press the collar into the beaker until the bottom is wetted at the surface of the nutrient solution.
  b. Make sure that the seedling's root is fully immersed in the nutrient solution
4. Using a sterile pipette, add 1 ml of N1 nutrient solution to the base of the seedling shoot at the top of the clone collar (Fig. 1B).
5. Secure the clone collar by placing 3 small pieces of paper tape on its outer top edge and along the side of the glass beaker (Fig. 1C).
6. To allow gas exchange, insert a sterile 1000 µl filtered pipette tip between the clone collar and inner surface of the beaker's spout until the opening of the pipette tip slightly emerges below the bottom of the clone collar (Fig. 1C). To increase the size of the tip's opening, remove approximately 0.75 cm from the small tip using sterile scissors before inserting into the beaker system.
7. To prevent light from reaching the roots, wrap aluminium foil around the outside of the glass beaker and secure it with paper tape.
8. Repeat the process (steps 1–7) for each seedling (for each validation experiment, 36 of the germinated seedlings were selected with sufficient root length to fit the hydroponic system).
9. Place the transplanted beakers in a sturdy 25.4 cm x 50.8 cm planting tray covered with a fitted plastic dome to reduce any shock due to decreased humidity in the seedlings’ new environment (Fig. 1D).
10. Place the trays into the growth chamber (16 h day, 8 h night, 23 °C, luminosity 210 µmol·m^−2^·s^−1^, relative humidity approximately 60%) and open dome ventilation slightly.
11. Remove the covers after 7 days.

### Cutting the primary root to encourage lateral root development

1. As soon as lateral roots begin to emerge from all seedlings (1–2 weeks after transplant), cut the primary root of each seedling to encourage lateral root elongation.
  a. In a sterile cabinet, briefly remove the clone collar (along with the seedling and filtered pipette tip) from the glass beaker.
  b. Using sterile scissors, cut the primary root 0.5 – 1 cm above the root tip and immediately below any emerging lateral roots (Fig. 2A). Take care to leave sufficient root length below the clone collar so that the root will be submerged in the nutrient solution when returned to the hydroponic system.

**Fig. 2:**
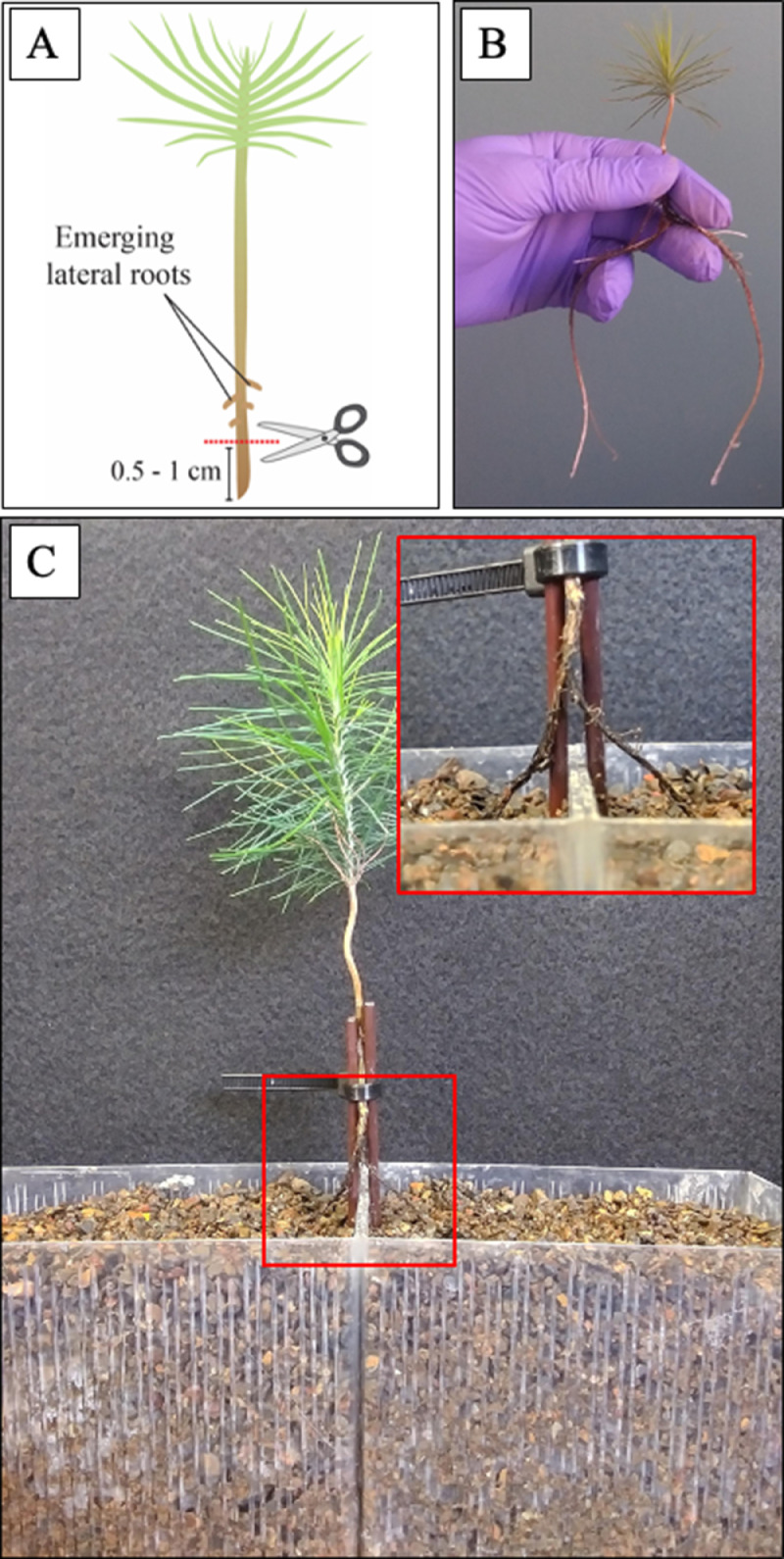
Split-root assays in *Pinus taeda* seedlings. A. The primary root tip of one-to-two-week-old *P. taeda* seedlings was removed with sterile scissors. Cuts were made once the first lateral roots emerged, and below them, removing 0.5 to 1 cm of the primary root. B. Eight weeks later, long enough lateral roots were visible, and plants were ready to be transferred into the separated split-root compartments. C. Each side of the split roots were placed into one of the compartments filled with substrate, with the seedling shoot directly above the adjoining walls of the planting boxes. The seedling shoot was secured in place by wrapping a sterilized cable tie around four plastic coffee stirrer supports, with the shoot in the centre of the four supports. The section of primary root from which all the lateral roots emerge was centred directly above the adjoining walls of the two planting boxes. Compartments were inoculated or not with ECM fungi depending on the experiment.
  c. Immediately place the seedling (along with the collar and pipette tip) into a new autoclaved 250 ml beaker containing 200 ml of fresh autoclaved N1 nutrient solution.
2. Repeat this process (step 1) for all seedlings.
3. Transfer aluminium foil coverings to new hydroponic beakers and return to planting trays in the growth chamber for an additional 6–7 weeks (total of 8 weeks in hydroponic systems).

### Maintenance of hydroponic seedling growth

Keep the seedlings in hydroponic beaker systems in the growth chamber for a total of 8 weeks after transplant.

1. Every 36 h, shake the trays by hand for 1 min to encourage oxygen diffusion into the liquid medium and prevent anoxia.
2. Every 2 weeks, refresh the nutrient solution in the beakers. (This may coincide with timing of cutting primary roots [see above] if lateral roots take 2 weeks to emerge for all seedlings.)
  a. For each seedling, place 200 ml of autoclaved N1 nutrient solution into a new autoclaved 250 ml glass beaker in a biosafety cabinet.
  b. Quickly transfer the clone collar containing the seedling, along with the filtered aeration pipette tip, to the new beaker and secure it with tape as before.
  c. Transfer the aluminium coverings to the new beakers and return beakers in planting trays to the growth chamber.

### Preparation of ECM fungal inoculants

  1. Four weeks before seedlings are transplanted into soil medium, inoculate liquid cultures of ECM fungus. The following steps describe this process for *P. ammoniavirescens* grown in Melin-Norkans (MMN) medium. For *H. cylindrosporum*, YMG medium was used instead of MMN. This process may be adapted to any ECM fungus, using an appropriate culture medium.
  a. Working in a biosafety cabinet, use a sterile cork borer (1 cm diameter) to cut a plug in the advancing edge of mycelia growing on MMN agar medium.
  b. Remove the plug with a sterile spatula and place into sterile 100 ml culture jar containing 50 ml of autoclaved MMN liquid medium.
  c. Cover the jars with sterile lid.
  d. Incubate the liquid culture jars in the dark at 26 °C for 4 weeks.
  2. On the day of seedling transplant, prepare fungal slurry inoculants in a sterile cabinet.
    a. Using a sterile spatula, remove the fungal thallus from the liquid culture jar and place it in an autoclaved 100 ml glass beaker containing 40 ml of autoclaved N1 medium. Gently swirl the thallus inside the beaker to rinse any excess MMN medium.
    b. Remove the rinsed thallus and place it in an autoclaved 100 ml glass beaker containing 80 ml of autoclaved N1 liquid nutrient solution.
    c. Using an immersion blender with a sterile grinding tip, grind the fungal thallus in the nutrient solution until no large fragments remain, but not long enough to completely homogenize the slurry (i.e., some small hyphal particles will still be visible floating in the liquid).
    d. After grinding, stir the slurry rapidly with a sterile spatula, and quickly divide evenly into two 40 ml aliquots in each of two sterile 50 ml glass beakers.
    e. Cap the glass beakers with sterile aluminium foil and keep at room temperature for inoculation of transplanted seedling roots.

### Transplanting split roots into separate compartments

After 8 weeks of hydroponic culture, lateral root growth will be sufficient that most roots are long enough to reach at least to the bottom of the 250 ml beakers (Fig. 2B). At this point, transplant the seedlings in separated split-root compartments with the following methods:

1. Rinse SafeT-Sorb™ substrate 3 times with DI water to remove very fine particles.
2. Sterilize planting boxes (12 cm × 8 cm × 8 cm; L × H × W; from Carmo A/S) and planting trays by soaking them in a 1/10 bleach dilution (1 part 6% bleach, 9 parts DI water) for 8 min. Then rinse boxes and trays 3 times in DI water to remove any residual bleach.
3. Place approximately 600 cm^3^ of rinsed, moist substrate in each box. The upper surface of the substrate should be no more than 0.5 cm below the top of each box.
4. On either side of the adjoining walls of each compartment, place two sterilized plastic coffee stir rods (cut to 12.5 cm) into the substrate directly against the wall of each compartment, approximately 1 cm from the centre of the adjoining edges. A total of 4 stir rods (2 in each compartment) can be used to provide support for the transplanted shoot (Fig. 1D).
5. Create a hole approximately 1 cm wide and 4 cm deep in the soil material and about 1 cm from the centre of the adjoining edge of each pot.
6. Wearing clean nitrile gloves, remove a seedling from its clone collar and carefully separate lateral roots into two approximately equal parts (Fig. 2C). For easier manipulation of roots, this separation was performed while roots were still submerged in the liquid nutrient solution in the 250 ml beaker.
7. Place each side of the split roots into one of the holes in the substrate medium, with the seedling shoot directly above the adjoining walls of the planting boxes. Make sure that any tertiary roots are planted in the same compartment as the secondary roots from which they originated.
8. Working quickly to ensure that roots remain moist, secure the seedling shoot in place by wrapping a sterilized cable tie around the plastic coffee stirrers supports, with the shoot in the centre of the four supports. The section of primary root from which all the lateral roots emerge should be centred directly above the adjoining walls of the two planting boxes (Fig. 2C).
9. For the non-mycorrhizal root segment:
  a. Pour 40 ml of N1 nutrient solution directly on the roots inside the hole in the substrate.
  b. Then fill the hole and gently compact the substrate around the roots by hand.
  c. Remove any substrate from the upper edges of the pot.
  d. All non-mycorrhizal root segments should be planted before mycorrhizal segments are inoculated and planted, to prevent cross-contamination of fungal growth from mycorrhizal to non-mycorrhizal sides of the split-root system.
  e. For the mycorrhizal root segments:
    Very carefully pour 40 ml of fungal slurry/N1 nutrient inoculant (from step 2 of “Preparation of fungal inoculants”, above) directly onto the roots in the open hole.
10. To prevent cross-contamination to the adjacent box, take great caution not to allow any slurry to spill or splash onto the adjacent root compartment or contact any roots above the substrate.
11. Gently compact substrate around the roots by hand.
12. Once all split-root boxes are planted in plastic trays, cover the trays with plastic domes and place them in the growth chamber. As described above, domes are meant to minimize transplant shock, and should be removed 7 days after transplant.

## Method validation

### Biomass is comparable between root halves in *Pinus taeda* split-root assays

**Fig. 3:**
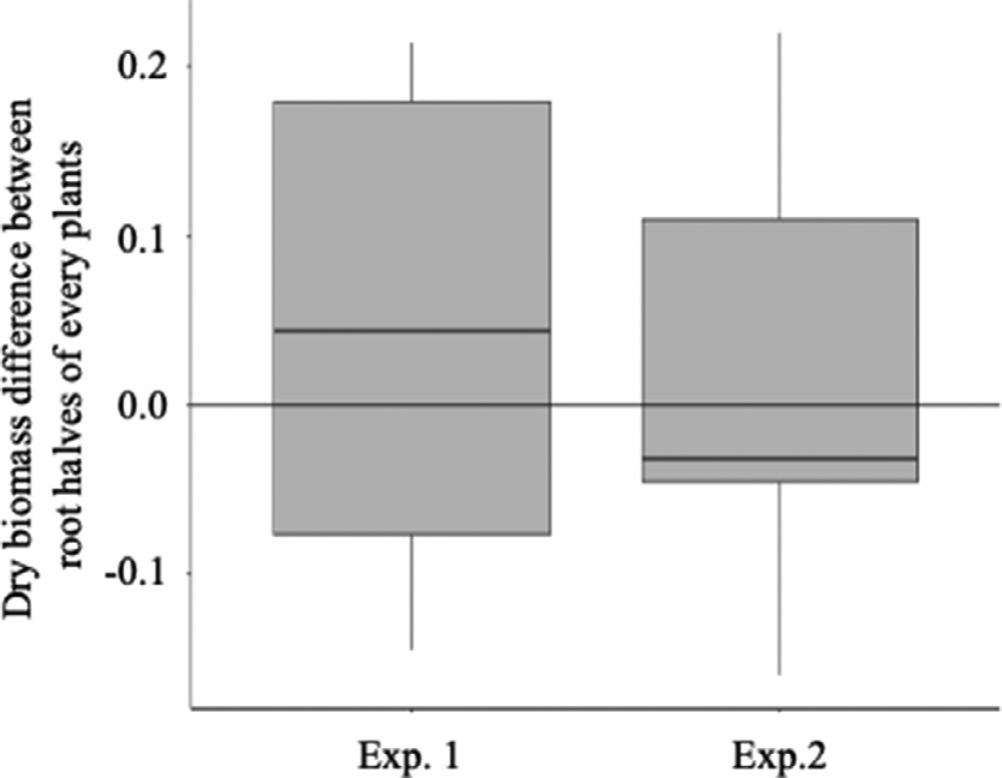
Biomass comparison between *Pinus taeda* root halves in split-root assays. Two independent experiments (Exp. 1 and Exp. 2) were performed. For both experiments, the roots of five to six plants were split as described in the *Method details* section and dry weight was recorded for each half of every plant. Difference in dry biomass between each half was calculated for every plant, and data presented here display an average of 5–6 plants. Paired-sample t-tests showed no significant difference in biomass between each root half, validating the split-root method for *P. taeda*.

To verify that split roots grew evenly in each compartment, two independent experiments were performed. Eight weeks after transplantation into the split-root systems, roots were cut directly above the substrate, and each root half was then removed separately from boxes. Roots were rinsed by submerging in DI water to remove any remaining particles of the substrate. Excess water was removed by gently pressing roots between two paper towels. Each root half was oven-dried at 70 °C for 5 days, dry weight was recorded, and the difference between each root half of every plant was calculated (Fig. 3). No significant differences in biomass were noted between each root half of every plant for both experiments. This result indicates that the split-root method we developed for *P. taeda* is reliable and that comparisons will be possible after any treatments applied to each half.

### Effective ectomycorrhizal colonization of *Pinus taeda* roots in split-root assays

**Fig. 4:**
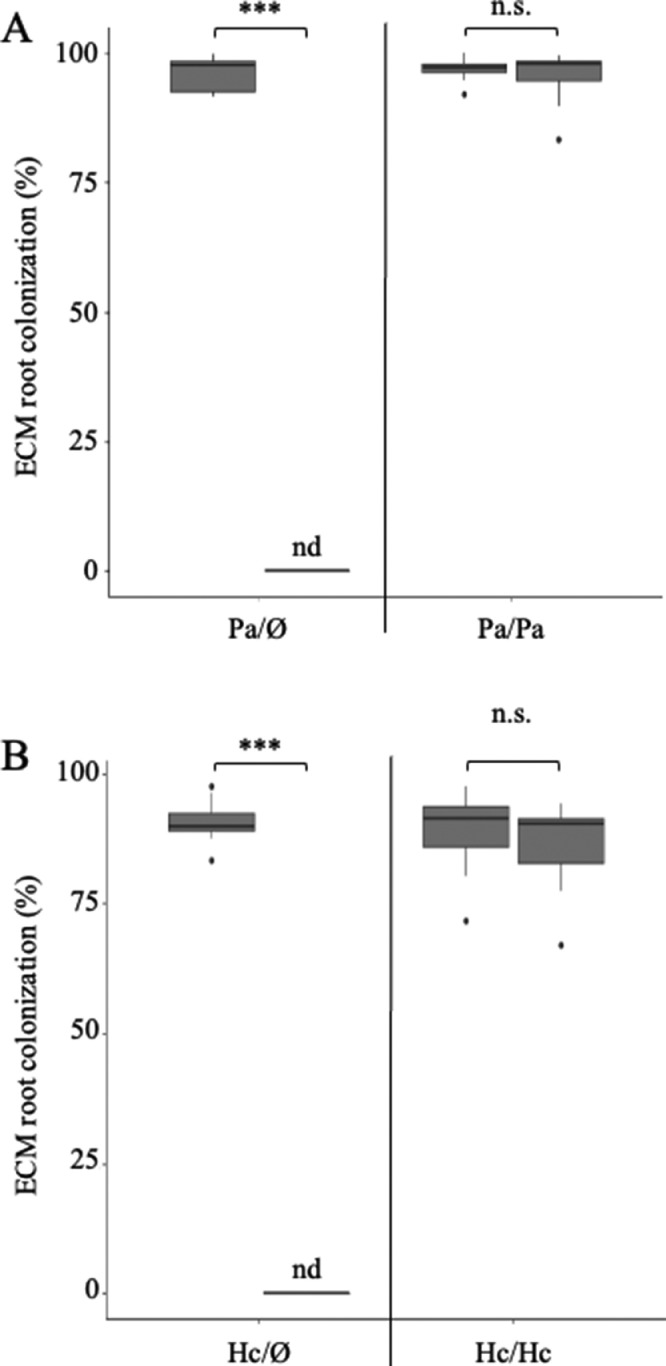
Ectomycorrhizal colonization rates of *Pinus taeda* seedlings in split-root assays. The colonization rate for each root half represents the percentage of short roots exhibiting ECM morphology relative to the total number of short roots in 8-week-old plants colonized by *Paxillus ammoniavirescens* (Pa, A), *H. cylindrosporum* (Hc, B), or kept non-inoculated (Ø, A,B). Colonization rate was compared between adjacent root halves using paired-sample t-tests. Asterisks (***) indicate significant differences between treatments (*P* < 0.0001) and “n.s.” indicates no significant differences between treatments (*n* = 5–6).

Being able to colonize only part of the *P. taeda* root system would allow comparisons between ECM and non-ECM roots in the same plant. Eight weeks after inoculation with the ECM fungi *P. ammoniavirescens* or *H. cylindrosporum*, roots were harvested, and colonization was recorded using a binocular scope (Fig. 4). Each short root tip was counted as colonized when a fungal mantle and the absence of root hairs were observed, or non-colonized if no fungal mantle was observed and root hairs were present. The percent colonization was determined as:

$$ECM\;colonization\%=\;\frac{colonized\;short\;roots}{total\;short\;roots}\;\times \;100$$

In plants where only one root half was inoculated with *P. ammoniavirescens* (Fig. 4A) or *H. cylindrosporum* (Fig. 4B), ectomycorrhizas were only observed in the inoculated roots. This result validates the absence of leakage from one root compartment to another, and indicates that this split-root assay can be used for ECM-related studies in *P. taeda*. Additionally, no significant differences in ECM colonization were observed in plants dually inoculated with *P. ammoniavirescens* (Fig. 4A) or *H. cylindrosporum* (Fig. 4B), indicating that the split-root approach developed for *P. taeda* did not alter the plant ability to interact with ECM fungi.

## Discussion

Here, we describe a simple method for developing split-root systems with young loblolly pine seedlings in symbiosis with ECM fungi using hydroponics. In loblolly pine, the single central primary root can present a challenge for establishing split-root experiments where the goal is to divide roots of the same plant into two equal segments. Here, primary root growth was inhibited by cutting the tip immediately after the emergence of lateral roots. Equal and uninhibited lateral root development was then allowed by freely suspending the roots in hydroponic solution. After 8 weeks, the lateral roots were long enough to divide equally into separated compartments (Fig. 2B) and inoculate with ECM fungi.

The split-root system described here is a simplified design intended to validate the efficacy of this method for seedling root development, transplant, and inoculation. No variable treatment conditions were added to adjacent roots, aside from inoculation. The absence of significant biomass difference found between paired roots (Fig. 3) implies that roots of the same plant were physiologically similar in adjacent halves with the same treatment following transplant. For experiments that may employ this method with additional treatment combinations in adjacent root compartments, this physiological similarity provides a control for the comparison of any observed treatment responses in paired roots. Similarly, colonization rates were not different between root halves with the same inoculation (Fig. 4A & 4B), indicating that this method allows control of the mycorrhizal status of adjacent roots in respect to treatment conditions in separate compartments.

In combination with carefully designed treatment applications, this method may be useful for researchers interested in studying systemic loblolly pine responses to ECM fungi and soil environmental conditions. Indeed, split-root systems have been used in the past to explore ECM interactions with other tree species, employing a variety of techniques to determine nutrient transport, host tree carbon allocation, and competitive interactions amongst fungal species [2]. For example, a split-root system was used with 3- to 4-year-old beech trees (*Fagus sylvatica*) to determine spatial distribution of host photosynthates and fungal nitrogen in ectomycorrhizae [16]. Another experiment with *Pinus muricata* elaborated on a similar design by including different ECM fungal species in adjacent root compartments to study the impact of nitrogen access on indirect competition between fungi for host carbon resources [9]. Although the timeline of this setup was similar to ours, using a hydroponic approach in glass beakers might help in evaluating and controlling the formation of lateral roots. *Pinus sylvestris* and *Pinus contorta* have also been used in mycorrhizal split-root experiments to study the effects of soil pH on host carbon translocation to ECM symbiont [7]. Consequently, it is possible that the described method could also be applied to these and other pine species, but this should be verified by trials with hydroponic growth. This method has been optimized for split-root development of loblolly pine and is intended to be adapted with variations of other techniques, as described above, to study resource allocation and local versus systemic responses to soil conditions in ECM loblolly pine roots.

Supplementary material *and/or* additional information [OPTIONAL]

Table S1: Recipe of the N1 medium used for hydroponics.

## CRediT authorship contribution statement

**Benjamin D. Rose:** Conceptualization, Methodology, Investigation, Visualization, Validation, Writing – original draft. **Hannah E.R. Frank:** Conceptualization, Methodology, Investigation. **Kevin Garcia:** Conceptualization, Resources, Writing – review & editing, Supervision, Project administration, Funding acquisition.

## Declaration of Competing Interest

The authors declare that they have no known competing financial interests or personal relationships that could have appeared to influence the work reported in this paper.

## Data Availability

No data was used for the research described in the article.

## Appendix Supplementary materials

Image, application 1
