# Supplementary material for: Development of split-root assays for loblolly pine (Pinus taeda L.) seedlings to study ectomycorrhizal symbioses
Source: MethodsX. 2023 Feb 5;10:102046. doi: 10.1016/j.mex.2023.102046 (PMC9939713; doi:10.1016/j.mex.2023.102046)
Supplement: Supplementary file 1 [file mmc1.docx]

**Table S1: N1 medium for hydroponic growth**

| **Stock solution** | **Final concentration needed** | **Volume (ml) of stock solution**  **for 1 liter N1 medium** |
| --- | --- | --- |
| Ca(NO_3_)_2_ [0.5 M] | 0.2 mM | 0.4 |
| KNO_3_ [1M] | 0.6 mM | 0.6 |
| KH_2_PO_4_ [1M] | 0.2 mM | 0.2 |
| MgSO_4_ [1M] | 1 mM | 1 |
| KCl [1M] | 0.2 mM | 0.2 |
| Iron (III) citrate [1%] | 0.5 ml/l | 0.5 |
| Micronutrient stock solution* | 0.2 ml/l | 0.2 |
| Thiamine** [10 mg/l] | 10 µl/l | 0.01 |

Adjust the pH to 5.5 with Ca(OH)_2_ or H_2_SO_4_

Autoclave at 120°C for 20 min

*see Table S2 for micronutrient solution

** add Thiamine only after autoclaving

**Table S2: Micronutrient stock solution for N1 medium**

| **Compound formula** | **Mass of compound**  **for 1 liter micronutrient solution** |
| --- | --- |
| MnSO_4_, H_2_O | 3.08 g |
| ZnSO_4_,7H_2_O | 4.41 g |
| H_3_BO_3_ | 2.82 g |
| CuSO_4_,5H_2_O | 0.98 g |
| Na_2_MoO_4_,H_2_O | 0.29 g |

Autoclave at 120°C for 20 min
